# Consigning Technology Acceptance to History. Introduction

**DOI:** 10.1007/s00048-026-00447-7

**Published:** 2026-03-09

**Authors:** Fabian Zimmer

**Affiliations:** Fachgebiet Technikgeschichte, TU Berlin, Berlin, Germany Straße des 17. Juni 135, 10623

## Consigning Technology Acceptance to History

The idea of “technology acceptance” exerts a continuing appeal in public debates, policy advice, and social scientific research on issues such as infrastructure projects, NIMBY-ism, participatory planning, the role of prosumers in socio-technological transitions, or in more general assessments of public attitudes towards innovation. Thus, in recent years, pressing issues such as the expansion of energy infrastructure in the transition towards a CO2-neutral energy system (especially power lines, wind and solar farms), or various aspects of digitalization such as autonomous vehicles, smart homes, or the introduction of large language models, have been discussed in terms of the “acceptance” of these technologies. “Acceptance” has thus appeared as a factor required in successfully mastering the Grand Challenges of our time—and corresponding governance subfields such as “acceptance management” have emerged (cf. for instance Bögel et al. 2018).

The appeal of “technology acceptance” persists despite a long-standing critique, which reaches back as far as the earliest formulations of the concept. In the 1970s and 1980s, the conflicts and controversies especially around nuclear energy, but also around genetic engineering, pollution, or new information technologies made clear that public acceptance of technology could no longer be taken for granted in industrial societies. It is at precisely this point that explicit concepts of “technology acceptance,” the “public acceptance of technology,” or the “social acceptance of technology” were first formulated by social scientists, and widely debated in the public and political spheres of Western democracies.

Already in these early debates, the concept of “technology acceptance” was criticized mainly on two grounds. The first of them was the vagueness of the term “technology,” which can refer to wildly different objects and thereby makes “technology acceptance” too blunt for an analytically or empirically valuable description of the differentiated attitudes and emotions people have towards technology (cf. Renn 1981). Accordingly, social scientific surveys of public attitudes to technology have repeatedly found a pervasive ambivalence towards “technology” *tout court*, while attitudes towards different types of technology vary greatly (cf. Renn 1981; Renn & Zwick 1997; acatech & Körber-Stiftung 2018). The second line of criticism refers to the top-down perspective embodied in the term “acceptance,” which naively assumes acceptance to be the normal attitude and attributes a passive role to consumers and the public, while resistance, reluctance, or even ambivalence towards a technology are marked as deviant and in need of correction. As Ortwin Renn has put it in an overview of a quarter century of social scientific acceptance research, this research was “initially set up to understand people’s seemingly irrational way of reacting to technology and to use this understanding to make corrections to perception” (Renn 2005: 36; cf. Aitken 2010; Batel et al. 2013).^1^

Approaches to “technology acceptance” do not seem to have moved far from this stance since. In 2011, at the height of the heated debates around the German nuclear phase out after Fukushima and the major railroad project Stuttgart 21, the German National Academy of Science and Engineering (acatech) issued a position paper, in which the full dilemma of measures towards increasing “technology acceptance” in democratic societies becomes apparent: even if technology communication is not meant as an “ad-hoc-instrument for the ‘procurement of acceptance’” *(“Akzeptanzbeschaffung”*—in scare quotes in the original!) but should rather foster “technological literacy” (*Technikmündigkeit*), the Academy nevertheless assumed, as given, a disquieting “phenomenon of *insufficient* technology acceptance, which is often perceived as a location factor inhibiting innovation” (acatech 2011: 9, my emphasis). Likewise, also historians of technology continue to casually use phrases like “insufficient technology acceptance” for describing past attitudes towards technological change.

In the light of this critique and the persisting appeal of notions of “technology acceptance,” this special issue makes the case for consigning “technology acceptance” to history—in both senses of the phrase. In joining the choir of critics described above, we argue on the one hand that notions of “technology acceptance” as an analytical tool for describing people’s attitudes towards technology should be left behind. On the other hand, we stress that “technology acceptance” nevertheless needs to be taken seriously from a historical perspective. We propose here to examine “technology acceptance” from the perspective of the history of discourses and knowledge, viewing it not as an analytical tool but as a discourse and a set of practices inscribed with specific power dynamics. Through case studies focusing on northwestern Europe since the middle of the twentieth century, this special issue traces how discourses of “technology acceptance” have shaped controversies over new technologies and technological choice.

## Concepts of “Technology Acceptance”

The term “technology acceptance” is used here as a shorthand for a number of related concepts such as “public acceptance of technology” or “social acceptance of technology.” It is also the most direct translation of the German “Technikakzeptanz.” However, all of these terms have their own conceptual histories as well as public trajectories.

The concept of “public acceptance of technology” appears to have originated in the US-American debates surrounding the foundation of the Office of Technology Assessment in the early 1970s.^2^ It was then most prominently used in the context of energy and risk debates of the 1980s, especially in debates around nuclear power (cf. Inhaber 1982; Nealey et al. 1983; Williams & Mills 1986). In this respect, the concept is very similar to the German “Technikakzeptanz,” which also developed around the same time and stood in close relation to the contested debates around nuclear power (cf. Renn 1981). Accordingly, in English publications by the Wissenschaftszentrum Berlin (WZB)—a central player in the German debate—“public acceptance of new technologies” is used as the translation for “Technikakzeptanz,” alongside “technology acceptance” (cf. Petermann & Thurn 1986; also published in Williams & Mills 1986). The term “social acceptance of technology” seems to have been used in a similar way but less frequently in these debates.^3^ Since then, however, it has gained popularity in the context of social scientific research on renewable energies in the last twenty years (cf. Wüstenhagen et al. 2007). Finally, “technology acceptance” has been closely related with the “Technology Acceptance Model (TAM)” developed by industrial engineer and MIT Sloan School of Management graduate Fred D. Davis since the 1980s for managing the user acceptance of new information technologies (cf. Davis 1985, 1989; Davis et al. 1989; see also the self-historization in Davis & Granić 2024). While the term “technology acceptance” is also used in reference to public attitudes towards technology, the TAM thus emphasizes the experience and behavior of individual technology users more specifically.

**Fig. 1:**
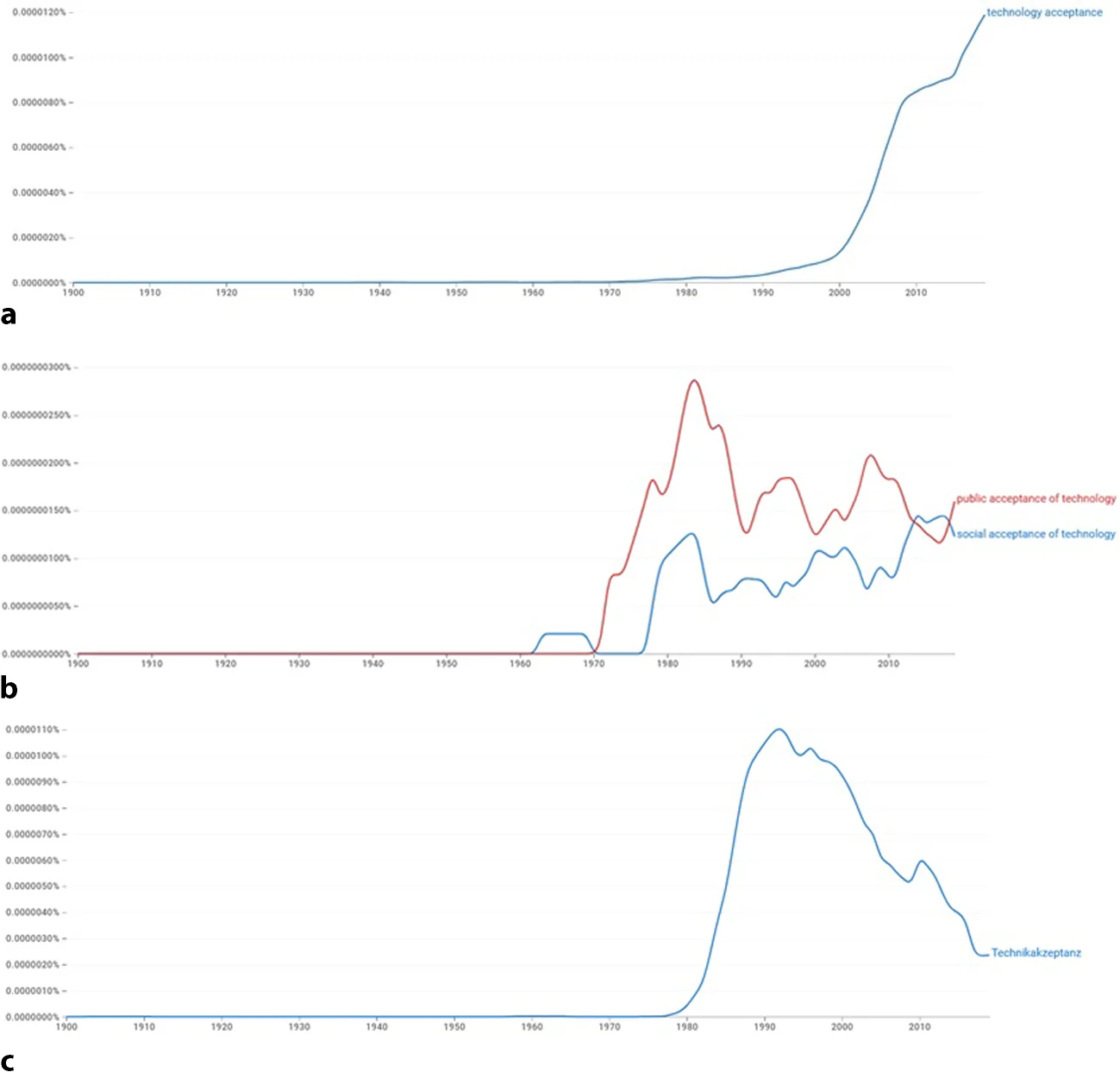
Google n‑grams for “technology acceptance,” “public acceptance of technology,” “social acceptance of technology,” and “Technikakzeptanz,” 1900–2018

What all these concepts have in common is that they gained traction during and after the 1970s—as a brief look at the corresponding Google n‑gram graphs can demonstrate (see Fig. 1). There is good reason to assume that this is no coincidence. Rather, debates on “technology acceptance” and explicit concepts of “technology acceptance” as a mode of social self-reflection appeared precisely at the point in time when public acceptance of technological innovation stopped being the prevalent norm in Western industrial societies. As numerous historians have highlighted, the period “after the boom” (Doering-Manteuffel & Raphael 2008) marks the waning of a “high modernist” consensus (Herbert 2007), which placed great trust in the capacities of technological innovation to produce economic and social progress (cf. van der Vleuten et al. 2017; Hänseroth 2013; Scott 1998) and sidelined more ambivalent voices (cf. Rieger 2003). Around 1970 at the latest, this consensus became increasingly contested, under pressure from the new social movements, and—with regards to technology especially—the environmental and antinuclear movements as well as from the oil price shocks of the 1970s (cf. Kupper 2003).

With this increased contestation of new technologies and a growing demand for democratic participation, “technology acceptance” now became a major concern especially for political and economic decision-makers in Western democracies. It was especially in the context of the Cold War that innovation in science and technology held a central place in ensuring competitiveness in the confrontation between the Eastern and the Western bloc (cf. Oreskes & Krige 2014). Against this backdrop, securing economic prosperity by maintaining an innovation-friendly culture was seen as essential in the competition between the blocs. It is thus no coincidence that the so-called “PANTs-project,” an early research project on the “public acceptance of new technologies,” was initiated at the 1982 G7 summit at Versailles, in order to stimulate the economic vigor of Western economies (Williams & Mills 1986).^4^ This example thus points to the importance of “technology acceptance” discourses as means of governance: “technology acceptance” was made a topic of debate and an object of research by stakeholders in controversies over technological choice as a means of gaining and maintaining support for specific decisions within democratic systems.

**Fig. 2:**
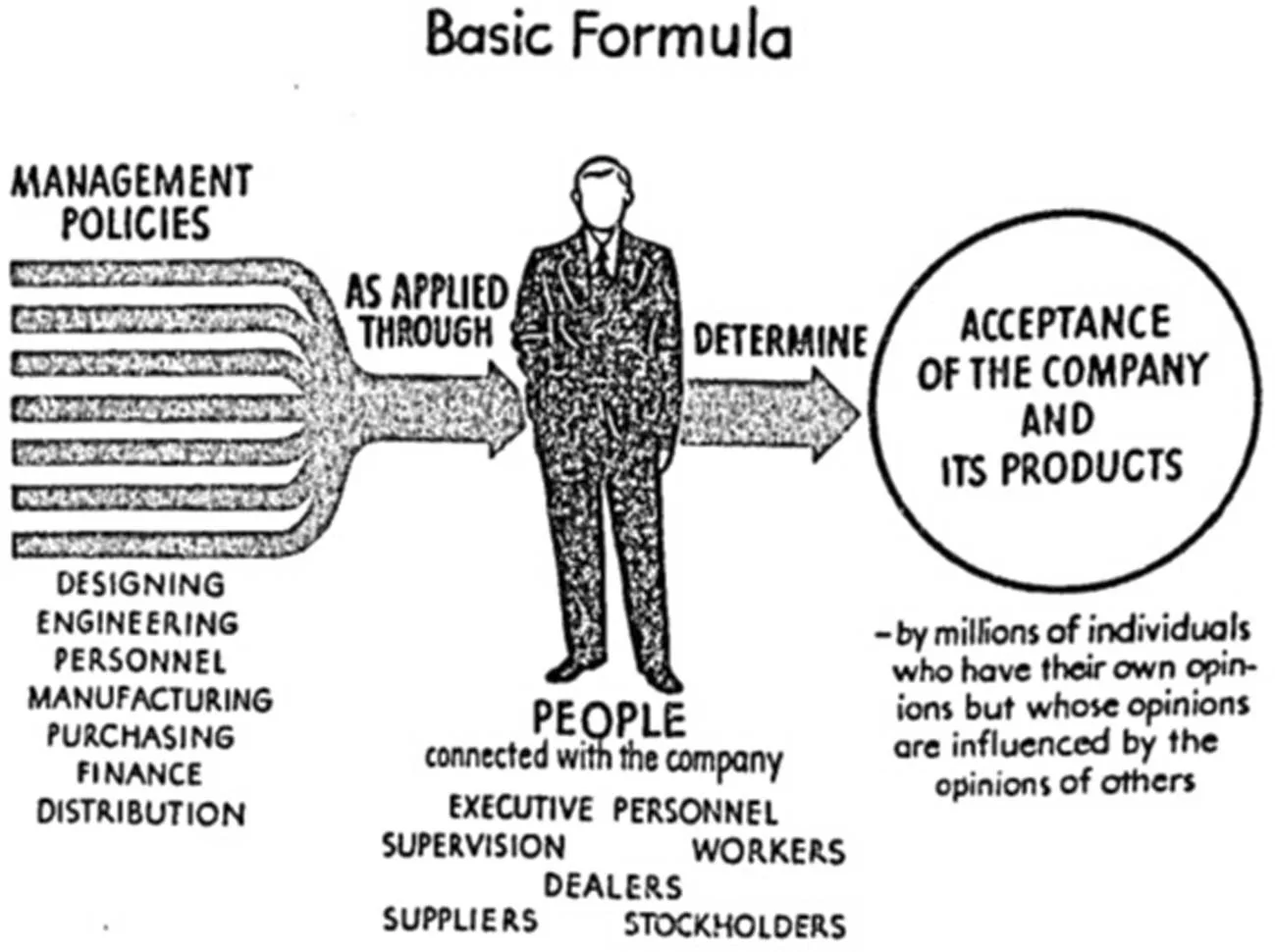
The “Basic Formula” of public relations as presented by Paul Garrett, director of public relations at General Motors, reproduced from Lindström (1958: 26)

While it is clear that the public debate and the explicit theoretical reflection on “technology acceptance” date back only to the 1970s, there is also a longer history behind concepts of “technology acceptance.” For instance, the public relations and management literature burgeoning since the middle of the twentieth century urged companies and managers to not only invest in advertising specific products to increase sales, but to influence and manage public attitudes in a much broader sense (cf. Ewen 1996). The goal of public relations activities now lay in attaining a wide “acceptance of the company and its products” (cf. Fig. 2).

**Fig. 3:**
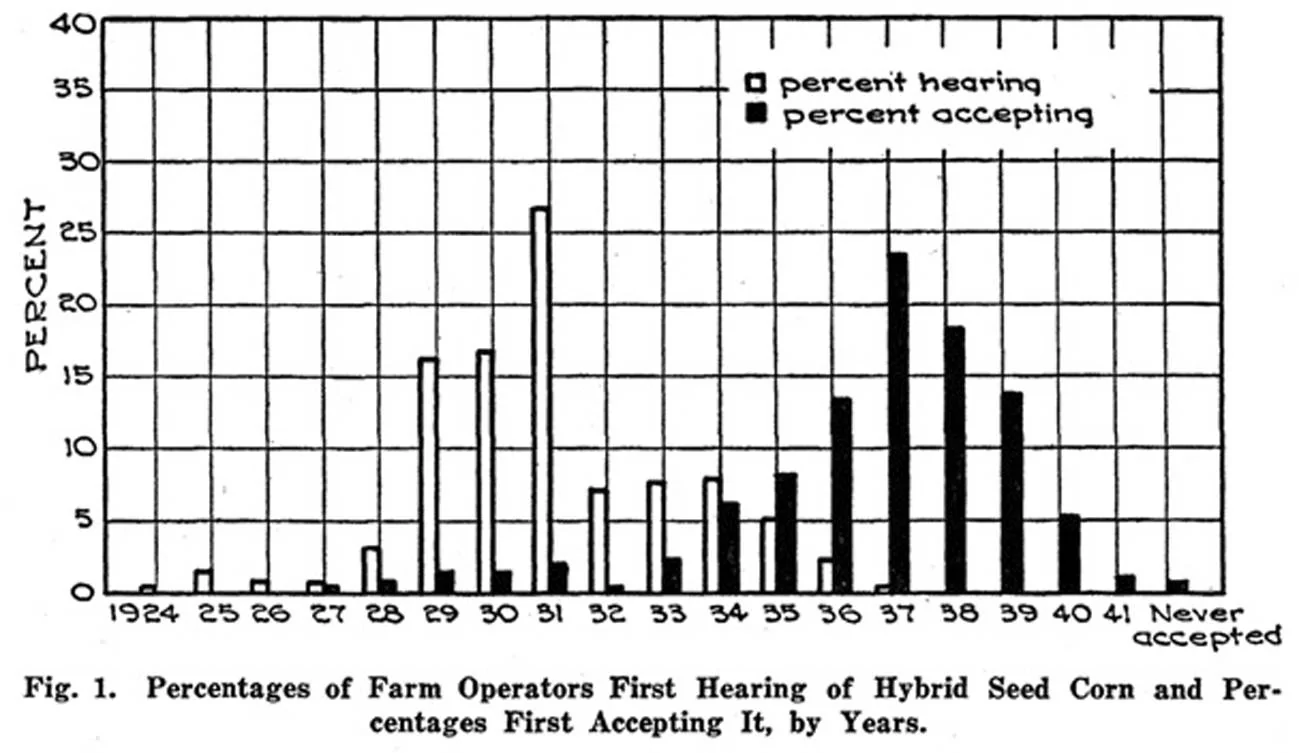
“Acceptance” of hybrid seed corn, as presented in a pioneering diffusionist study by Gross & Ryan (1943: 17)

Likewise, in the postwar period, notions of “acceptance” became closely linked with the then burgeoning discourse of “innovation” (cf. Popplow 2020). Explicitly or implicitly, “acceptance” formed the last stage in the various linear models of innovation which were formulated during this period (cf. Godin 2006). As such, “acceptance” played an important role for instance in social scientific studies on the diffusion of technological innovation since the 1940s (for instance Gross & Ryan 1943; cf. Valente & Rogers 1995). In these studies, however, “acceptance” was largely synonymous with the “adoption” of specific technologies, that is: it figured as a measure of the proportion of a specific group actually using a given technology (cf. Fig. 3). It is only since the 1970s that social scientific concepts of “technology acceptance” emphasize the *attitudes* people have towards technology. One might thus hypothesize that concepts of “technology acceptance” underwent a process of psychologization since the 1970s—a hypothesis which can perhaps be seen in line with the “psychoboom” of the period (cf. Tändler 2011). It is in this period, that “technology acceptance” acquired a pronouncedly normative and diagnostic meaning. No longer merely a matter of the use or non-use of a given technology, “technology acceptance” now became tied up with much larger questions, such as the limits of public participation, the state of democracy, or the future of society.

## Acceptance and the History of Technology

The history of technology as a discipline is not external to this field. Rather, historians have taken and continue to take up varying positions within the resistance-acceptance-spectrum. Indeed, one could even argue that the constitution of the history of technology as a discipline at the turn of the twentieth century was deeply embedded in the struggle of the emerging engineering profession for its own social and cultural acceptance as well as for the acceptance of the technologies it designed.^5^ Today, as the discipline has become an established part of the historical (rather than the engineering) disciplines, the spectrum has broadened: while someone like Andreas Malm turns historical arguments into activism in his book *How to Blow Up a Pipeline *(Malm 2021), others like energy historian Richard F. Hirsh see one of the purposes of the history of technology in helping policymakers “overcome nontechnical barriers” and address “social impediments to acceptance of novel technologies” (Hirsh 2011: 20; see also Hirsh & Jones 2014).^6^ These positions certainly mark two extremes, whereas most historians would probably subscribe to an intermediate position (cf. Högselius 2021). Such a position was already articulated by historian Ulrich Troitzsch at a 1987 conference on “History of Technology and Technology Acceptance,” where he argued for an intensified “historical acceptance research,” which promised to make a contribution to the then emerging field of Technology Assessment and to be a means of “rationalizing the discussion” (*Versachlichung der Diskussion*, Troitzsch 1988: 39; cf. also the other contributions in the same volume).

Indeed, since the 1970s, historians have investigated numerous public conflicts around new technologies or infrastructure projects (cf. for instance Linse et al. 1988 or Aschwanden et al. 2022), and analyzed discourses of technophobia (cf. Sieferle 1984) and technophilia (cf. Segal 1985) as well as their more ambivalent variants (cf. Nye 1994; Rieger 2005). Furthermore, the engagement with simplistic notions of “technology acceptance” has brought historical and sociological thinking about innovation and technological choice to a new level of theoretical sophistication. Arguing against innovation- and industry-centered accounts, which place the agency of users and the public only at the accepting end of a linear model of innovation, new concepts have emerged, which highlight the “social construction of technology” (Bijker et al. 1987), the “cultural appropriation of technology” (Hård & Jamison 2005), and the “co-construction of technology and society” (Oudshoorn & Pinch 2005), or which see contested technologies such as nuclear energy as “public technology” (Trischler & Bud 2019). These efforts have thus contributed to the criticism of simplistic notions of “technology acceptance” by highlighting technological choice and the impact of users and the public (cf. Edgerton 2006). However, these approaches, as essential and deserving as they undoubtedly are, tend to downplay the power relations in public conflicts over innovation. They tend to overlook the formative discursive power that a concept such as “technology acceptance” has held and continues to hold in these debates.^7^

## On the Contributions to this Special Issue

In light of these tensions—between the critique and the continuing appeal of framing issues of technological choice in terms of “technology acceptance,” as well as between a well-justified focus in historical research on the agency of users and the public and the persistent formative power of acceptance discourses on historical reality—this special issue proposes to historicize “technology acceptance” by examining it from the perspective of a history of discourses and knowledge. The contributions collected here do not aim to reiterate the entire technology acceptance debate of the past fifty years. They are not primarily concerned with finding out why it is that people “resist” novel technologies or what the preconditions are for “acceptance,” nor do they want to highlight in particular the ambivalence in attitudes towards new technologies or investigate how users and the public have indeed shaped technologies. What we envision here, is a history of concepts, discourses, knowledge production, and knowledge circulation *about* what we call “technology acceptance” today, and of how this knowledge and these discourses have been put into practice in conflicts over technological choice. The papers in this special issue thus present case studies in which “technology acceptance” does not figure as an analytical tool, but rather as a historically situated discourse and set of practices. In keeping with approaches from the history of knowledge (cf. Sarasin 2020, 2011; Füssel 2021), the papers are concerned with concepts, actors, media, and practices which have shaped discourses of “technology acceptance” in twentieth-century northwestern Europe.

The four papers collected here were first presented at the international workshop on *Histories of technology acceptance in the 20th century *at the Department for the History of Technology at TU Berlin in October 2023, generously funded by the Deutsche Forschungsgemeinschaft (DFG, German Research Foundation)—529533640.^8^ Their case studies follow the constitution and use of acceptance discourses in a roughly chronological order and through different fields of technology.

The first two papers take a close look at the practices of fostering public technology acceptance through corporate public relations in the energy industry. Focusing on the postwar period since the 1950s, these two papers trace how players in technological modernization conceptualized and promoted acceptance *before* explicit notions of “technology acceptance” emerged. In his close reading of public relations films by the Swedish State Power Board, Vattenfall, *Fabian Zimmer* traces an adaptation discourse which pervaded not only the PR work of the State Power Board, but took advantage of a wide societal and political discourse in postwar Sweden, which framed conflicts over modernization and technological choice as matters of individual adaptation. Zimmer’s paper furthermore highlights the importance of emotions in acceptance discourses, as he traces the discursive establishment and maintenance of a standard narrative about the emotions at play in processes of technological change. In the second paper, *Christian Götter* offers a case study of the British Central Electricity Generating Board and its local efforts to promote acceptance at the Oldbury-on-Severn nuclear plant from the late 1950s on. In a thick description based on local newspapers, Götter traces the “mundane practices by which technology acceptance was produced and maintained on the ground.” These practices aimed at demonstrating the accountability, transparency, prestige, and familiarity of nuclear power and the local installations and, as Götter demonstrates, they were flexibly adapted over several decades—according to the power plant’s local life cycle much more than according to the national or transnational debates on nuclear energy.

The following two papers then concentrate precisely on the debates which have shaped the discourse of “technology acceptance” since the 1980s, focusing on case studies from West Germany and Switzerland. The paper by *Johann Meyer* investigates discourses around the introduction of Electronic Data Processing in German-speaking Protestant churches from the late 1960s to the early 1990s. His paper questions established narratives about the “technophobic church” and demonstrates in a close reading of publications and internal church documents, how this topos served specific discursive purposes in inner-church disputes about power and hierarchy. In particular, Meyer demonstrates how computer-savvy pastors could mobilize the topos of the “technophobic church” as an “emancipatory term” in an act of self-empowerment. In the final paper, *Thomas Lettang* returns to debates on energy, but from a different perspective than the opening papers. Lettang investigates the “intellectual and political history of technology acceptance” through political and social scientific debates on energy saving in West Germany. Following technology acceptance debates “from above and below,” Lettang traces the emergence of the concept of “social compatibility” (*soziale Verträglichkeit*) and investigates practices of participation and representation in citizens’ letters. He thereby shows how both social scientists and citizens were not so much concerned with the contemporary dystopian visions of an “atomic state” or a “calorie state,” but rather with questions of distributive fairness and the steering capacity of the state.

Read together, the papers presented in this special issue thus aim to make a dual contribution: firstly, on an empirical level, they shed light on the deeper history of current notions of “technology acceptance,” by presenting rich case studies in which discursive practices of “technology acceptance” become tangible, as well as the power dynamics enacted through acceptance discourses. Secondly, on a theoretical level, by historicizing a debate in which historians of technology have played an active role, they contribute to a better understanding of the history of the history of technology as a discipline and thus to a better understanding of the epistemic conditions under which we operate today.

## Footnotes

1 All translations by the author.

2 The term figures in the appendices to several committee documents around the creation of the Office of Technology Assessment Act of 1972 found in the Google Books corpus. Committee on Rules and Administration (1972: 74) thus claims in a “Summary of Pro and Con Arguments for an OTA” that with the implementation of the Office of Technology Assessment a “sound, technically-based climate of public acceptance of a new technology could be established in advance instead of waiting for a defective technology to become a political issue.” On this context, see also Doil (2022).

3 For instance, in a strategy paper from the Japanese Ministry of International Trade and Industry (Matsuda 1981), in the UNESCO-edited journal *Impact of science on society* (Mihai 1983), or in the more popular book by Ramo (1983).

4 The (perceived) importance of technology acceptance for maintaining economic prosperity was also highlighted by Andie Rothenhäusler in his paper “Framing or genuine debate? Technikfeindlichkeit as a discourse topic of the Bonn Republic in the 1980s” at the *Histories of technology acceptance in the 20th century* workshop in October 2023 at TU Berlin.

5 Adelheid Voskuhl raised this important point in her paper “Engineers’ social and technological acceptance in the Second Industrial Revolution” at the *Histories of technology acceptance in the 20th century *workshop.

6 For an early example along the same lines, see Skowronnek (1934).

7 For instance, in their portrayal of nuclear power as a “public technology,” Trischler & Bud (2019) rely on several cases of public relations activities (such as exhibitions) by industry and government. In their effort to demonstrate how much the public has shaped technology, a reflection of how the public itself *has been shaped* is lacking in their account.

8 The individual papers as well as this introduction have profited greatly from the contributions by all workshop participants. I would like to thank all speakers and commentators, especially the ones whose contributions are not represented in this volume: Aybike Alkan, Chase Galis, Karena Kalmbach, Panagiotis Kazantzas, Antonis Kechrimparis, Christopher Neumaier, Ortwin Renn, Andie Rothenhäusler, Tiia Sahrakorpi, Heidi Schweickert, Aristotle Tympas, Adelheid Voskuhl, and Heike Weber. Furthermore, I would like to thank the students who participated in my seminar *Geschichte der Technikakzeptanz im 20. Jahrhundert* at TU Berlin in the winter semester 2023/24 and who have helped sharpen many points raised in this volume: Esme Buden, Yaroslav Koshelev, Antonia Mittelbach, Colin Murphy, Melena Schneider, Clara Schütz, and Lea Stengel.
